# The problem with self-management: Problematising self-management and power using a Foucauldian lens in the context of stroke care and rehabilitation

**DOI:** 10.1371/journal.pone.0218517

**Published:** 2019-06-19

**Authors:** Simon Fletcher, Stefan Tino Kulnik, Sara Demain, Fiona Jones

**Affiliations:** 1 Faculty of Health, Social Care and Education, Kingston University and St George’s, University of London, London, United Kingdom; 2 School of Health Professions, University of Plymouth, Plymouth, United Kingdom; 3 Bridges Self-Management Limited, London, United Kingdom; Plymouth State University, UNITED STATES

## Abstract

Self-management is a concept which is now firmly established in Western healthcare policy and practice. However, the term remains somewhat ambiguous, multi-faceted and contentious. This is evident in stroke care and rehabilitation, in which a self-management approach is increasingly adopted and advocated, yet interpreted in different ways, resulting in contradictions and tensions around control, responsibility, power and discipline. This paper aims to further our understanding of tensions and contradictions in stroke self-management, by critically examining contemporary self-management practices. We use a Foucauldian theoretical lens to explore the various power dynamics in the operationalisation of self-management, in addition to the complexity of the term self-management itself. Conducting a secondary analysis of interview and focus group data from the Self-Management VOICED study, supplemented with analysis of relevant documentary evidence from policy and practice, we describe the multiple aspects of power in operation. These include rhetorical, hierarchical, personal and mutual forms of power, representing interweaving dynamics evident in the data. These aspects of power demonstrate underlying agendas and tacit and explicit understandings of self-management which exist in clinical practice. These aspects of power also give insight into the multiple identities of ‘self-management’, acting as a simultaneous repressor and liberator, directly in keeping with Foucauldian thinking. The findings are also consistent with Foucault’s notions of bodily docility, discussions around governance and biopower, and contemporary discipline. Our analysis positions self-management as a highly nuanced and complex concept, which can fluctuate in its conceptualisation depending on the structures, routines, and the individual. We encourage healthcare professionals, policymakers and commissioners in the field of self-management to reflect on these complexities, to make transparent their assumptions and to explicitly position their own practice accordingly.

## Introduction

The self-management concept made its first symbolic entry into the arena of stroke care and rehabilitation a decade ago, when the United Kingdom (UK) national clinical guideline for stroke included the term ‘self-management’ in a passage on self-efficacy training [1]. Since then, stroke self-management approaches have burgeoned. A considerable number of clinical studies have since been completed, generating a growing evidence base [2–4]. Topical reviews and influential opinion pieces in the medical and rehabilitation literature now actively promote the inclusion of self-management into stroke care pathways [5, 6], and the latest UK national clinical guideline for stroke now includes a dedicated self-management topic section, recommending that

People with stroke should be offered self-management support based on self-efficacy, aimed at the knowledge and skills needed to manage life after stroke, with particular attention given to this at reviews and transfers of care [7].

However, despite an emerging evidence base, stroke self-management remains a rather ambiguous, multi-faceted, and contentious term.

### Origins of self-management

Lorig and collaborators [8] at Stanford University, California, are widely acknowledged as pioneering the self-management concept during the 1990s in the group-based Stanford Chronic Disease Self-Management Programme (CDSMP). In the late 1990s the Stanford CDSMP was launched as the Expert Patient Programme in the UK National Health Service (NHS), embedding self-management in UK health policy [9–11]. Morden and colleagues [12] outlined this development during the UK Labour government, which held power until 2010, and the subsequent coalition and Conservative governments have continued this trajectory. Under the banner of ‘Compassionate Conservatism’ and the ‘Big Society’, the latest policy directions have increasingly incorporated ‘self-management’ in notions of community involvement, encouraging both public and voluntary sector through collaborations such as the ‘Realising the Value’ programme [13].

Kendall and Rogers [14] describe these developments as ‘state sponsored’ forms of self-management, juxtaposing them with lay practices of self-care that have existed for centuries, and with the more recent self-help movement [15]. They contend that, while the CDSMP originally followed a humanitarian and client-centred ethos, state sponsored self-management is implicitly based on a professional compliance model, assuming that the best self-management practices are those developed by experts in positions of authority, and optimal outcomes are achieved through patient compliance. This also encourages a moral imperative for individuals to look after their own health and avoid ‘risk’ behaviours [16, 17]. This moral burden is growing through the combination of increasing life expectancy and the rise in chronic health conditions, which results in economic strain on the healthcare and welfare system, as evidenced in this recent statement by the World Health Organisation:

Given the right guidance and support, empowered people can address damaging health behaviours and/or challenges in their environment that prevent healthy lifestyles. Supporting self-management will be critical for many countries where ageing populations and the growing burden of non-communicable disease means that there is ever greater demand for health services [18].

Therefore, state sponsored self-management becomes a social obligation [12, 14, 15, 19]. This is, however, offset by the additional development of self-care and self-help practices, which have originated from natural activity, and re-appropriation of healthcare by lay people through access to health knowledge and self-help technologies. These activities make up a form of self-management that is driven by people with disabilities and chronic conditions, rather than medical and economic concerns, aligning with aspects of the social model of disability and ambitions of equity, voice, engagement and emancipation [14, 15].

### Complexity

The composite origins of self-management give some insight into the complexity of the concept, which is reflected in the difficulty in categorically defining the term. Authors acknowledge that there is not one universal definition of self-management [20, 21], but widely cited definitions include the following by Barlow and colleagues [22]:

[Self-management is] an individual’s ability to manage the symptoms, treatment, physical and psychosocial consequences and lifestyle changes inherent in living with a chronic condition. Efficacious self-management encompasses ability to monitor one’s condition and to affect the cognitive, behavioural and emotional responses necessary to maintain a satisfactory quality of life, thus, a dynamic and continuous process of self-regulation is established [22].

Similarly, the clinical research literature conceptualises self-management interventions as complex interventions, comprising several interacting ingredients and mechanisms of action. Related and overlapping terminology and concepts, such as patient education, problem-solving, goal-setting, coping, adaptation, person-centred care, shared decision-making, etc., each warrant definition and conceptualisation in the particular context of application [20]. This can lead to considerable confusion over the conceptualisation of clinical interventions, when interventions which were not explicitly designed and labelled as self-management, are regarded as such on the basis that they align with the theoretical mechanisms of action that underpin self-management. Examples of this were given in a recent meta-review of stroke self-management interventions by Parke et al. [4], which included reviews of rehabilitation therapy interventions on the basis that these incorporate problem-solving, decision-making and goal-setting processes. Conversely, Lorig and Holman [8] have suggested that some interventions labelled as supporting self-management do not meet the criteria of what constitutes a self-management intervention.

### Self-management in stroke care and rehabilitation

From the mid-2000s, the idea of tailoring the self-management approach to people with stroke and other neurological conditions has attracted interest [23]. Individuals with neurological conditions often experience considerable levels of disability, which may be due to physical and/or cognitive impairments, leading to care and support needs in daily life. In addition, progressive neurological conditions present a general trajectory of gradual deterioration and mounting care needs in the future [24]. The provision of self-management interventions for people with stroke and other neurological conditions therefore faces considerations that are specific to these groups, and which need to be reflected in the design of interventions [24]. Firstly, a common-sense, or naïve, interpretation of ‘self-management’ as ‘managing by myself/on my own’ can equate self-management to ‘functional independence’ and ‘economic self-sufficiency’. This may introduce tension when individuals depend on practical and financial assistance for activities of daily living, potentially in the long-term [25, 26]. Secondly, cognitive and/or communication difficulties in people with neurological conditions are common, but the self-management approach relies on cognitive processes (e.g. problem-solving) and dialogue between patient and healthcare professional [26, 27]. Thirdly, with some degenerative neurological conditions, there is the potential for disease progression leading to sudden, unpredictable changes (e.g. multiple sclerosis), which introduces additional challenges for a self-management approach [24, 28].

In the stroke self-management literature, it is possible to discern three principal aspects of the underlying understandings of self-management. First, self-management after stroke may be viewed as a strategy for optimising secondary stroke prevention, for example in the studies by Hill et al. [29], Sajatovic et al. [30] and Damush et al. [31]. Here, the focus is on improving stroke survivors’ adherence to medical management (prescribed medication and compliance with healthy lifestyle advice), to achieve improvements in stroke risk factors (hypertension, hypercholesterolaemia, hyperglycaemia, obesity, etc.) and reduce the risk of further stroke or transient ischaemic attack.

Second, following the dominant trends around maximising dose and intensity of therapeutic physical activity and exercise, self-management may be understood as an approach for increasing stroke survivors’ general physical activity levels, or adherence to specific rehabilitative self-exercise [5]. Examples are studies by Mansfield et al. [32] and Moore et al. [33], in which accelerometer devices were used to document stroke survivors’ physical activity and arm exercise. Much of this work is based on the use of innovative technology to reinforce self-management goal-setting through individual motivational bio-feedback, but also enabling rehabilitation professionals to oversee stroke survivors’ actual (as opposed to self-reported) performance and adherence to physical activity and exercise plans [34].

Third, self-management may be regarded as a strategy to enhance stroke survivors’ activities of daily living, participation in society and quality of life. These aspects are articulated, for example, in studies by Wolf et al. [35], Tielemans et al. [36] and Harwood et al. [37]. In this context, the rationale for self-management revolves around understanding stroke as an acute event with resulting long-term consequences, which include participation restriction and social isolation. While acute healthcare and rehabilitation provision in the first weeks and months following stroke is often comprehensive and intense, many stroke survivors experience a sense of abandonment after discharge from formal services [26, 38, 39]. Supporting stroke survivors in their self-management skills may therefore bolster individuals’ resilience and resourcefulness in rebuilding their lives after stroke in the long-term.

Issues reflected in these three strands of applied stroke self-management revolve around adherence to/compliance with health professionals’ advice; the morality of making healthy lifestyle choices; the transition after discharge from statutory services; or indeed the withdrawal of formal service provision and its substitution with patient self-management. These points invite reflections on tensions and contradictions in the field of stroke self-management, relating to control and responsibility and undercurrents of power and discipline. While these tensions have been described in some of the more critical academic commentary on self-management in general [14, 15, 19], these aspects have not yet been explored specifically with respect to self-management in stroke care and rehabilitation. This study therefore aimed to further our understanding of tensions and contradictions in the field of stroke self-management by examining contemporary self-management practices. Our research questions were ‘How can we make sense of tensions and contradictions around control, responsibility, power and discipline in stroke self-management?’ and ‘What are the resulting practical implications for operationalising stroke self-management?’

## Method

We conducted a secondary analysis of interview and focus group transcripts from Self-Management VOICED [40], a large qualitative research study of self-management in stoke, diabetes and colorectal cancer funded by the Health Foundation, an independent charity in the UK. We re-interpreted these data from a Foucauldian perspective, applying an associated thematic analysis and linking this with relevant documentary evidence from stroke policy and practice.

### Theoretical approach

We applied the work of Michel Foucault, with particular reference to his concept of governmentality [41–43]. The shift towards neoliberal healthcare models, the implementation and development of self-management approaches and the subsequent discord between perceptions, represent multiple realities and sophisticated power systems. The way that self-management may not only represent a constraining product of an intelligently dominant social context but also a legitimate and amoral development of patient choice is strongly consistent with Foucault’s approach to power structures. In a removal from more traditional theories of power, in which dominance, suppression and hierarchy are relatively fixed, Foucault suggests that power can go beyond oppression:

[Power] induces pleasure, forms of knowledge, produces discourses. It needs to be considered as a productive network which runs through the whole social body, much more than a negative instance whose focus is repression [41].

It is possible to see parallels in the implementation of self-management approaches. Taking charge of one’s illness and engaging with a personal expertise which transcends traditional healthcare induces pleasure and forms knowledge [19] whilst simultaneously contributing to the continuity of wider systems of control.

### Data

We utilised all data from Self-Management VOICED which related to stroke only (excluding data from diabetes and colorectal cancer). This comprised anonymised transcripts from four focus groups and two individual interviews with stroke survivors (37 stroke participants in total), and nine individual interviews with healthcare professionals and commissioners of NHS services (the latter refers to individuals who are appointed and responsible to plan and commission NHS healthcare services for their local area). An overview of participants is given in Table 1. Data were collected with the aim to identify (1) the outcomes of self-management support important to people with stroke, commissioners of self-management services and health professionals; and (2) areas of thematic similarity and disparity in the self-management outcomes of importance across these groups. Data collection took place between June 2014 and December 2015. Focus groups and interviews were conducted in three UK locations (Southampton, London and Leeds), representing urban and rural settings with ethnically and socially diverse populations. Participants were purposively recruited to ensure compliance with the inclusion criteria, and to maximise diversity in terms of time since diagnosis, age and ethnicity for stroke survivors, and professional expertise for health professionals and commissioners. Included were individuals over the age of 18, who were living (stroke survivors) or working (health professionals and commissioners) within a 50-mile radius of the locality. People with aphasia were included if possible, and the facilitator/interviewer employed supportive communication strategies to enable their participation. Stroke survivors were excluded if they had a stroke less than three months previously. Health professionals were included if they had expertise in working with people with stroke and/or self-management. Commissioners were invited if their remit included commissioning services for people with stroke and/or self-management. Depending on the group of participants, focus group and interview guides varied slightly in terminology, but broadly asked the same questions. Questions asked for: an introduction, either to the condition (stroke survivors) or job role (health professional, commissioner); participants’ understanding of self-management; and important outcomes of self-management. All participants gave written informed consent prior to data collection. Ethical approval was granted from the Research Ethics Committee at University of Southampton, including secondary analysis of anonymised data [40].

**Table 1:** Overview of participants.

**Stroke participants**	**Data collection method**	**Number of participants**	**Gender**
	Focus group (FG1)	10	Female: 5 Male: 5
	Focus group (FG2)	11	Female: 8 Male: 3
	Focus group (FG3)	8	Female: 3 Male: 5
	Focus group (FG4)	6	Female: 3 Male: 3
	Interview (I1)	1	Female
	Interview (I2)	1	Male
**Healthcare professionals**^a^	**Pseudonym**	**Gender**	**Professional role**
	HCP1	Male	General Practitioner, Chair of a Cardiovascular Clinical Commissioning Programme
	HCP2	Female	Stroke Clinical Lead Speech and Language Therapist, Acute Stroke Unit and Early Supported Discharge Service
	HCP3	Female	Psychologist, Community Rehabilitation Service
	HCP4	Female	Speech and Language Therapist, Community Rehabilitation Service
	HCP5	Female	Stroke Clinical Lead Occupational Therapist, Early Supported Discharge Service and Community Rehabilitation Service
	HCP7^b^	Female	Occupational Therapist, Community Specialist Stroke Services
	HCP12	Male	Physiotherapist, Early Supported Discharge Team
	HCP17	Female	General Practitioner, Self-Management Champion
	HCP19	Female	Nurse Practitioner, Self-Management Clinical Programme Manager

FG, focus group; HCP, healthcare professional

^a^All healthcare professionals took part in individual interviews.

^b^Non-consecutive numbering due to sub-sample from the larger study including other clinical conditions.

In addition to interview and focus group data, relevant stroke policy and practice documents were purposively selected to situate these data within a wider national framework. These documents were identified by co-authors FJ, SD and STK from their knowledge of the wider academic and grey literature in stroke and self-management; and through a targeted search of electronic databases, evidence gateways (e.g. NHS Evidence) and websites of stakeholder organisations in the field of stroke and self-management (e.g. Stroke Association, King’s Fund, Health Foundation) using the search terms ‘self-management’, ‘self-care’, ‘individualised healthcare’, ‘neoliberal’ and ‘stroke’.

Documents were selected purposively to represent views and directions in the self-management topic as it relates to stroke care and rehabilitation from government, NHS/clinical practice, and the voluntary/charitable sector within the UK national context. The documents included in analysis are listed in S1 Table.

### Analysis

Transcripts and documentary evidence were stored in NVivo software. SF and STK then read and re-read the data for familiarisation and conducted open coding in parallel, to identify sections that spoke to the presence and influence of narratives of power in self-management practice. Concepts developed through an iterative process of coding, cross-referencing and debriefing within the group of co-authors. SF and STK first undertook a line-by-line approach to coding. Following the process of open coding, they were able to identify and then establish four main areas in which power was enacted in the interviews and focus groups. These areas include rhetoric, hierarchy, personal empowerment and reciprocity and serve to emphasise how power can be regarded as both productive and restrictive. In the interests of rigour and consistency SF and STK then cross-referenced their initial findings and shared these with the wider research team (FJ, SD), subsequently returning to the data twice more. Through this process the research group were able to develop a framework which enabled the classification of the coded data in the first instance, and a comparison with the remaining transcripts and documentary data in the second. Policy and practice documents were also imported into NVivo, and relevant sections within these documents were linked to codes and concepts from the analysis of participant data. Whilst the data were specifically interrogated for thematic areas which surrounded power dynamics, ownership of condition, practitioner-patient relationships and ambiguity, it was possible to eventually identify the four primary concepts which were consistently identifiable and then ran through the course of the analysis. In the presentation of the findings, these have been labelled rhetorical power, hierarchical power, personal power, and mutual power.

## Findings

Our analysis focused on issues and dynamics of power resident in the daily practice of stroke self-management as enacted by people with stroke, stroke clinicians, rehabilitation professionals, health and social care practitioners, and commissioners of stroke rehabilitation services. We identified several aspects of power, which emerged from interview and focus group data. These focus around notions of rhetoric (rhetorical power), hierarchy (hierarchical power), personal empowerment (personal power) and reciprocity (mutual power). These aspects illustrate the complexity of self-management as evidenced by those individuals who inhabit the social space of stroke self-management practice. In the following, we provide illustrative anonymised quotes to support our analysis. Pseudonyms denote whether the speaker was a health care professional (HCP), or a male (M) or female (F) stroke survivor and which focus group (FG) they took part in.

### Rhetorical power

It became clear that ‘self-management’ is not an easily understood term or concept. ‘Self-management’ may be colloquially interpreted at face value as managing alone, but this interpretation barely captures the many facets of self-management as it is discussed in healthcare literature and policy. Power can be identified in the specialisation of the term ‘self-management’, which links to a specialist body of knowledge and is therefore a privileged term. Those individuals who are familiar with and have some understanding of the concept and history, and insight into the meanings and agendas behind ‘self-management’ are in an advantageous position over those who lack this specialist knowledge. Mostly stroke professionals will benefit from this, as their professional status provides access to specialist knowledge; but it is also possible for stroke survivors or lay members of the public to acquire this knowledge and power, for example through training programmes such as the Expert Patient Programme, or through engagement in voluntary sector organisations that operate in the self-management marketplace. The distinguishing feature of this rhetorical power is that the speaker uses the language of ‘self-management’ to pursue multiple and potentially conflicting agendas, which may include realising cost savings, reducing formal service provision, increasing patient influence in service design or improving patient choice.

The quote below reflects a sophisticated professional understanding of self-management from this physician and commissioner, who describes three aspects of the concept:

I can run from the fact the person who has a minor self-limited illness managing that themselves without having recourse to trying to seek professional advice […] I think there’s also (sighs) I think it’s a different concept but probably is meant when people say it, is that the person with a chronic long-term condition is the expert in their own condition, and the danger is that I think we’ve over-medicalised it and we’ve taken control of a condition that is somebody’s. […] Self-care can also be more general as well in terms of how do you encourage people to live a healthier lifestyle, you know, biggest one is how do you help people stop smoking. We can help people stop smoking but that’s something they do for themselves […] So how do we help people eat more healthily, take more exercise, that’s another element of self-care as well, isn’t it? So for me it [self-management] can be one of three things that are interrelated but not necessarily the same.

(HCP1)

There was also evidence of a recognition among clinical staff that self-management has become a terminological product of a particular healthcare context. One clinical psychologist comments: ‘I suppose I’m aware it’s quite a buzz word at the moment’ (HCP3), calling into question the extent to which the practitioner accepts or recognises its status in its original sense.

The way in which self-management was described to focus group participants also offered insight into the rhetorical presentation of the term, and the way its representation is required to fluctuate depending on audience. The facilitator describes self-management below:

Well, sometimes healthcare professionals will use that to describe the things that a person can do to help their recovery and to help manage their own health, and if you’ve got a long-term condition like stroke then you’ll be doing that anyway […] you don’t see health professionals very often now so what you are doing the rest of the time when you’re not seeing them those are the things that we would class as things that you’re doing to help your own health and well-being.

(FG1-Facilitator)

Whilst this appears measured, pragmatic and inherently patient-focussed, a practitioner offers a seemingly contrasting account of the professional ‘act’ which self-management necessitates:

So self-management starts right from the time you get into the patient’s house and saying hello, you know? You start straightaway, because it is all about our language as well. […] And if they say I am going to do this for you today, rather than them saying I am going to do this for you, I can probably ask the patient how they have been, and what did you manage to do, and so it is about the language as well.

(HCP12)

### Hierarchical power

There was also evidence of more recognisable forms of power, i.e. an absolute influence that is exerted in a unidirectional manner by an overwhelming authority and its representatives. This is the type of hierarchical power that is very difficult to challenge by individuals who are involved in daily self-management practice (professionals, people with stroke and their family members and caregivers). In the data, this power was mainly represented through economic decisions, such as the commissioning or de-commissioning of stroke rehabilitation and support services, rationing of hospital days and appointments with stroke professionals, and the funding that is available or lacking for staffing (with respect to both numbers and skills/seniority of staff) and specific mechanisms/services to support self-management.

The care plans described below provide an insight into the pressure under which healthcare staff operate, and the potential for the mismanaged or inappropriate utilisation of self-management:

So the surgery that I work at uses [self-management] care plans that we've devised at the CCG [Clinical Commissioning Group], but I know that they are not dedicated care plan users that have rolled them out, because they feel like they've been pressurised into using them. Rather than using them for everybody with a long-term condition as and when they come to the surgery, or, you know, identifying them at the right time, rather than just rushing through it and getting it done just to tick boxes. So that's how it goes against the grain.

(HCP19)

Whilst the following statement offers an example of the everyday pressures which therapists respond to:

We’ve got just enough staff to react in a timely way and that’s what commissioners want to see as well, they want to see us reacting really quickly and running around doing early supported discharge and dragging people out of hospital. I think a lot of the focus in this area had been on that front end of the pathway, so that sort of filtered down here. We run round and react rather than actually think about what happens in the longer term.

(HCP3)

### Personal power

There is an implication here that there is little scope for self-management under such a regulatory environment, as it requires longitudinal investment, the impact of which is never immediately visible. However, this can be responded to, and directly challenged by stroke survivors’ ownership of their life experience. This ownership manifests power through the personal experience of stroke and its consequences (as opposed to professionals who are unlikely to have had this experience); or power from the experience of caregiving from the perspective of stroke survivors’ family members and friends. This notion of being an expert from personal experience is reflected in the following comment by a stroke survivor:

I went on a course a few weeks ago, called the Expert Patient Course, and it opened my eyes, really. No disrespect to the medical people at the hospitals and all that, but we're the experts. You'll not find any doctors there who've had a stroke and recovering from a stroke.

(FG3-M1)

Another, more implicit example of personal power is evident in a focus group discussion among stroke survivors (FG2). Here, the facilitator opens by encouraging participants to ‘talk about how you manage your health and your wellbeing and how you help yourself after your stroke.’ The wording introduces the ideas of ‘health and wellbeing’, which create a medical context, and ‘helping yourself’, which introduces the issue of functional independence. But rather than following these leads, participants talk about doing enjoyable activities, socialising, and keeping busy, as ways of keeping going and preventing frustration, anger and low mood:

F1: I was just going to say it’s good food […] And obviously good friends and socialising, getting out and about doing stuff and exercise […]

F2: Go dancing and swimming. Go to the club […]

F3: Yes. I do feel very well. I feel I look after myself and go out. And I try and get up in the morning as early as possible and I enjoy doing lots of things like, I don’t know, I love doing the choir and I enjoy, just enjoy life really […]

M1: When I had a stroke, you think too much, as you can talk and you’re really angry as well. So, there’s loads of emotions going on. And if you do things, you’re so angry. And then most people here, for us that chat it’s great […] But you need people, you need people.

M2: You do need people around all the time.

All: Yes.

### Mutual power

There were also examples of mutual power in the data. These describe a realisation of self-management practice that leads to mutually beneficial, empowering arrangements between those who represent formal stroke services (stroke clinicians, rehabilitation therapists, health and social care professionals, formal caregivers, etc.) and those who represent users of stroke services (people with stroke and their next of kin, family members and friends). This mutual power may be viewed as representing reciprocity and the humanitarian aspect of self-management, which is underpinned by values of equity, voice, engagement and emancipation, as in this comment from a stroke clinical lead occupational therapist:

I would very much love to see patients, particularly stroke, empowered to know what they need to do to help themselves, both on a physical and a psychological level, and know to move forward. So, the information and the skills that we have passed onto them. And then for the service, I would like to see people coming back, as they perhaps sooner say, ‘Actually I need this, but I don’t need that, I want this from you, but I don’t want that’.

(HCP5)

This ethos of mutual power is also reflected in the enrolment of people with stroke in the delivery of self-management support interventions. This stroke survivor, for example, talked about his motivation to train as an Expert Patient Programme tutor himself:

I want them [referring to programme participants] to be able to fulfil everything in their life that they wanted to do beforehand and, if possible, give them that direction to go forward. […] I mean, it's been hard work doing all these things that I do, and you've got to push yourself to the limits some days, you know, but my achievement is not, I'm going to be a tutor. It's what they achieve. […] It gives me a good feeling in here.

(FG3-M1)

### A confluence of power dynamics

While our analysis has identified these aspects of power in isolation, in interviews and focus group discussions there was an interwoven confluence between and amongst them. To demonstrate this complexity, the quotes below highlight the respective forms of power within the text itself, e.g. (rhetorical), referring directly to the preceding passage. The following excerpt from a focus group discussion (FG2), for example, reflects subtle aspects of personal, mutual and hierarchical power. Personal and mutual power are reflected in participants’ accounts of what–in their experience–supports their self-management, and this includes seeking support from the doctor and having things to do, such as attending a Stroke Club where talks are given. The participant's remark that the Stroke Club has been ‘packed up now’ illustrates hierarchical power, representing their lack of influence on the provision of a valued community resource.

Facilitator: So if you are good at managing your health, how would you know that things are going well with your health? What things tell you that you are managing your health well? […]

M1: You go to the doctor […] If it’s not well so prescribes a pill or something (personal), (mutual). […]

F5: I used to belong to the Stroke Club, close to us then. And that was all right, but they packed it up now, you see, I’ve actually got nothing to do now (hierarchical).

Facilitator: And when you went to the Stroke Club, was that something you found was helpful in terms of managing your health?

F5: Yes. There were things we did and people that would come and do different things […] once a month. It was like groups, for instance, would come and give a talk. […] And that was fine. (personal), (mutual) But I’ve actually got nothing to do now (hierarchical) […]

Facilitator: And doing things is something that you find helpful in terms of managing?

F5: Yes. (personal)

The following statement contains elements of rhetorical, mutual and personal power. Here, a health professional reflects on her work with one particular stroke patient:

I think for him, because he couldn’t avoid being a part of life, so it kind of made him interested, it made him keen and it made him curious to work out what did and didn’t work for him (personal), and I think also it probably helped because I think just because of who he was and his personality, he quite liked the fact that I think our relationship developed, it felt like quite an equal, collaborative one (mutual). So, at first he was very passive but actually there was a big part of him that was used to being quite assertive and was used to that. So we could get to that part of him (rhetorical).

(HCP3)

Subsequent references within the same interview relate to hierarchical power:

I think it’s a challenge getting that prioritising and even though commissioners all want these people to live well with their conditions, actually when it comes down to brass tacks it’s all how many contacts are you seeing and when are you going to discharge this person and how are you going to make sure you see this person quickly and always end up going higher up the list (hierarchical). So you sort of think you’re all trying to do the right thing (mutual) but then a sideswipe comes in (hierarchical).

(HCP3)

The statement below incorporates rhetorical and mutual power, demonstrating how at the same time the practitioner performs an ‘act’ whilst making a genuine attempt to get the ‘real story’ out. This reflects the negotiating processes which occur between both parties when self-management is introduced. Power is both utilised independently and reciprocally here:

Okay, so I would go through and I think it [self-management] starts with a relationship thing anyway to begin with because it’s all getting on with people to begin with when they come in and engaging with them, helping them to understand that you are on their wave length and you are actually listening to them, you are understanding them, you listen for a minute or two with just the odd prompt to get the real story out (rhetorical), (mutual).

(HCP17)

This interplay between rhetorical and mutual power was even more pronounced in accounts of those stroke rehabilitation professionals who spend comparatively longer periods of time working and building rapport with people with stroke and their families, such as occupational therapists, speech and language therapists and physiotherapists. Here, professionals’ sophisticated understanding of the self-management concept and their ability to apply self-management rhetoric could be used to shape the relationship and interaction, to realise mutually empowering ways of working, or to negotiate professional or other agendas. In the following example, a therapist talks about her understanding of self-management:

I think it’s sort of totally what I believe in, so, really the people are experts of their own condition (personal), and I try and empower them with that knowledge. So really me not working as an expert, but sort of working alongside them really, sort of sign-posting them, building confidence, so that they have the confidence to take those steps, the reassurance that it is the right thing for them to be doing (mutual).

(HCP7)

In addition to this collaborative and empowering aspect of self-management, the interviewee then extends the self-management rhetoric to include an agenda of patient behaviour change, taking responsibility for one’s own health and making recommended health choices:

It is encouragement really, to sort of take responsibility for their own health, really. I find it quite a good window for behaviour change for a lot of people, they had quite a shakeup. So, they’ve known perhaps for some time that they should, you know reduce their weight, or reduce the alcohol or whatever. So, it is a real key time, and I guess it is just discussing those issues, […] what has happened to them, and supporting them to sort of make those choices, really (rhetorical).

(HCP7)

## Discussion

A Foucauldian interrogation of aspects of power identified in our data analysis has enhanced our understanding of self-management and emphasises the need to adopt a nuanced approach to its application. Beginning with rhetorical power, audiences are offered insight into the multiple ‘identities’ of self-management. The concept is, and should be, understood in different ways by different people and utilised throughout a variety of representative forms. While this might seem self-evident, it is worthy of attention under a Foucauldian approach. Going beyond simple notions of contextual perceptions (e.g. patient versus practitioner), the shifting focus we observe reflects the fluctuation of contemporary structures of discipline. It is possible here to observe circumstances in which social phenomena (such as self-management) offer individuals opportunity whilst directing their actions, thereby acting as a simultaneous repressor and liberator. We have presented a number of examples, in which the stroke survivor is given a sense of ‘ownership’ over their rehabilitation provision whilst remaining under the ultimate direction of a knowledgeable healthcare practitioner. Our findings have therefore identified that practitioners are able to both control the provision of care by engaging in a ‘performance’ when self-management is explained and administered; and that patients are able to assert their own rehabilitative and by extension individual independence. This is reflective of the mutual incentives evident in Foucault’s governmentality concept [41–43] and helps audiences to understand self-management from a perspective which incorporates both traditional doctor/patient dominance and more progressive forms of individual affirmation.

Hierarchical power exists throughout healthcare and is strongly embedded in both practice and ideology. Self-management is inherently affected by this, and this should be acknowledged as we approach its fluctuating ‘identity’. Wider context cannot be overlooked, although it is perhaps possible to regard self-management as a reflection of the Foucauldian modernisation of discipline. Self-management represents a contemporarily relevant response to the conventional power structures in healthcare as the approach avoids a number of the associated constraints. It is a general expectation that financial, managerial and commission-based pressures are all relieved when self-management is utilised, and this has multiple benefits. The power which exists in self-management can be viewed as productive in this instance. It is dispersed throughout the social body rather than simply enacted by one dominant, visible entity, however the hierarchical gaze remains in operation. As self-management eases financial burdens, the hierarchical sources of dominance are simultaneously appeased and diminished. The systemic and visible forms of discipline which would have been associated with a traditional patient/practitioner relationship should now be reinterpreted in a way which fragments previous notions of social control.

Personal power provides an opportunity to explore Foucault’s notion of bodily docility in a way which differs slightly from more conventional uses. Under the disciplinary society in which human bodies are a further representation of fragmented power, anatomy becomes a characteristic of social control. The pressure therefore to maintain a healthy functioning body is less overtly but in some ways more intensively exerted, as the body no longer belongs entirely to the individual. If a disruptive bodily event such as stroke occurs, the individual is in a sense relieved of the requirement to contribute to the continuity of this disciplinary activity, as the conditions under which they act are immeasurably altered. A person’s ownership of their life experience (personal power) is subsequently far more achievable after stroke as the disciplinary society does not accommodate such disruption, either explicitly or implicitly.

Mutual power is similar to rhetorical power in its incorporation of multiple levels and systems of control, yet the genuine reciprocity which is in evidence above encourages a more organically balanced utilisation of contemporary mechanisms of control. It is possible to explore the positive characteristics of self-management such as equity, voice, engagement and emancipation, and position them as indisputable strengths of the approach. Foucault’s discussions around governance and bio-power [41, 43] become particularly relevant here. The way in which self-management is an objectively positive influence over its users, providing a platform for affirmation of self-identity and control in a context in which their reliance on others is pronounced, whilst still acting as a governing and managerial entity which ultimately controls populations en masse, provides a working representation of what we see when we describe mutual power.

### Confluence of power in documentary evidence

The evidence of the confluence of power, the incorporation of two or more of these aspects of power, was strongly apparent in the analysis of the transcripts. Whilst this reaffirms the complexity of the systems of power which are in operation in stroke self-management, it also represents the (Foucauldian) dissolution of power throughout the social body, and the capacity of contemporary power for both mobilisation and restriction. This confluence of power is further reflected in documentary evidence. One example of this is the document ‘Self Care–A Real Choice. Self Care Support–A Practical Option’, which in 2005 described the UK government’s vision of ‘deploying the biggest collaborative resource available to the NHS and social care–patients and the public’ [10]. Using the term ‘self-care’ synonymously with ‘self-management’, the document contains many strands of the power dynamics we describe, in an example of highly sophisticated rhetorical power. (Of note, it is acknowledged that some authors use the terms ‘self-care’ and ‘self-management’ interchangeably, while others use these terms to denote different understandings or facets of a shared concept. The relationship between ‘self-care’ and ‘self-management’ is therefore more complex than semantic, although an interrogation of this in further depth is beyond the scope of this manuscript.) The case for self-management is made first through describing increased individual choice and control, which parallels a consumerist notion that choice in products is good (p. 2). Then, a list of potential savings and reductions in workload for health services is given (pp. 2–3). The argument then turns to support for self-care, making the case that people need to be supported in developing the skills for self-care (p. 8). There is a strong moral imperative underlying this, which is illustrated in a long list of things people can (or should) do, such as following a healthy lifestyle and becoming health literate (p. 8). There are also several examples in the document that align with mutual power, such as partnership working and the suggestion that services work with the public to realise integrated self-care ‘support’.

The use of the term ‘support’ in this context (e.g. ‘self-management support’ or ‘supported self-management’) warrants some attention. Today, the pervasive economic argument for self-management in health policy remains based on costs and burden of an ageing population and increasing prevalence of long-term conditions. The perception that self-management is a strategy for decreasing public service provision and placing more emphasis on personal responsibility may alienate service users and healthcare professionals alike. The word ‘support’ may be regarded as softening the rhetoric, making it more agreeable to those who take a skeptical or cynical view of the self-management agenda. Conversely, it may be interpreted as an appreciation of the fact that individuals often need to learn and develop self-management skills; and that some can require more support and/or practical assistance with this than others. This becomes particularly relevant in conditions such as stroke, where physical and/or cognitive impairments may lead to activity limitations and dependence on practical assistance and care in everyday life. Accordingly, the recommendations given in the UK national Clinical Guideline for Stroke consistently describe the provision of ‘help’ and ‘support’ with self-management (see pp. 113–114 in [7]).

Interestingly, the argument for self-management in stroke rehabilitation is not as firmly embedded in rhetoric about the economic burden of chronic disease as in more generic health policy documents; and it is not as openly presented as a deliberate strategy for reducing service use (possibly because there is generally little service provision after the acute and subacute stroke rehabilitation phase). Rather, self-management is framed as a strategy for dealing with the long-term consequences of stroke, and life after stroke in general. In some documents self-management is described alongside calls for more, or more consistent stroke rehabilitation service provision (e.g. [44, 45]), which mirrors the ongoing national conversation in the UK about inequity in stroke service provision in comparison with other health conditions [46, 47]. For example, ‘The NHS long term plan’ published in 2019 [48] calls for the promotion of ‘supported self-management’ to enhance out-of-hospital care and upstream prevention for people with different long-term conditions (pp. 15, 33), and at the same time promises higher intensity rehabilitation for people recovering from stroke in the community (pp. 64–65). In the document ‘Commissioning guidance for rehabilitation’ [49], self-management features prominently, but is linked to a vision of person-centred and empowering rehabilitation services. Here, rather than following the reasoning that self-management will relieve pressures on health and social care services, the economic argument for rehabilitation is that investment in rehabilitation will lead to cost savings in the mid- to long-term. In this respect, the concept of self-management conveyed in these documents aligns with the mutual and personal power aspects we describe. The question of how to realise self-management when individuals have high levels of dependency on others is resolved by referring to the role of families, carers and advocates, implying that the concept is then extended to, or transferred onto these individuals.

### Implications for practice

One of the criticisms of self-management is that it is embedded in political agenda [50]. However, precisely the fact that self-management is not only embedded in agenda, but also accommodates the pursuit of convergent, parallel and/or divergent agendas, may be part of its successful and widespread adoption in health policy and practice. In a Foucauldian sense, everything is essentially rooted in agenda and often financial imperatives. Rather than rejecting the concept of self-management on the basis of underlying agenda, we suggest that self-management can bring about positive developments, provided that the value base from which it is operationalised is made explicit.

Our analysis may contribute to the practical application (operationalisation) of self-management, in that it articulates the complexities of the dynamics that can come into play, firstly by demonstrating that self-management is not a simple or basic concept, but rather inherently nuanced and multifaceted. Our findings have highlighted the inadequacy of categorisations represented and evidenced in stroke self-management literature that range between seeking to promote healthy living, adherence to treatment regimens and managing life after stroke (or after discharge from statutory services). Professionals tend to situate their conceptualisation of self-management within a healthcare context governed by their responsibilities during the acute stages of care and the need to expedite discharge. Commissioners have a longer term gaze governed by the imperative for efficient use of scarce resources balanced with meeting the needs of their local population. Patients appear to be largely unaware of the morality of self-management and tend to move away from the term when articulating the benefits of the concept in relation to their needs for social participation and wellbeing. These differences could be partly explained by the temporal aspects of stroke self-management and the staged transition from periods of intense care and support to discharge, whereby patients naturally refer to outcomes relating to their own wellbeing, largely unaware and not influenced by targets about length of stay or discharge from statutory services. Our analysis has brought these differences into greater focus and positions self-management as a highly nuanced and complex concept, which can fluctuate in its conceptualisation depending on the structures, routines, and the individual. This in itself can be a worthwhile message to some clinicians and researchers who may seek understanding in a somewhat simplistic deterministic and reductionist worldview.

Second, we suggest that our analysis may enable individuals to reflect on their own conceptualisations and interpretations of self-management, and the power dynamics inherent therein; and therefore facilitate explicit articulation and positioning within this framework. In keeping with Foucault, rather than suggesting that one particular aspect emerges or should be viewed as superior, we are comfortable with the existence of multiple strands of power, which may align, run in parallel or diverge, depending on the social construction of self-management practices in a particular context. Having undertaken this analysis we would however recommend that practitioners, commissioners and policymakers should reflect on and make explicit their own position and how this might influence their practices in self-management. An example of this is seen in the document ‘Improving self management support’ published by the Scottish government in 2009 [51]. The principles of self-management, on which the document is based, are made transparent in its introduction and include the statement:

“Self management is not a replacement for services. … [It] doesn’t mean going it alone” Self management does not mean managing my long term condition alone. It’s about self determination in partnership with supporters. [51]

This declaration of underpinning understandings of self-management sets the tone for the remainder of the document. It explicitly addresses and counters a perception (or experience) on part of the reader that self-management initiatives provide political vehicles for de-commissioning of services, and instead emphasises a mutual and humanising approach in which people are ‘partners rather than recipients of care’ (p. 3). Similarly, from our own theoretical perspective and practical work, we ourselves view self-management as grounded in collaborative approaches, best represented by the mutual power aspect we have described, and we suggest that it should be operationalised accordingly. This links with participatory and co-production methods in the design and delivery of self-management interventions [52, 53]. Others have commented that individuals with chronic conditions can benefit from this emancipatory quality of self-management, even when they are unfamiliar with the notion of self-management as a concept itself (as many stroke survivors and lay members of the public are) [15]. The element of engagement, exchange and collaboration between individuals in the self-management arena is thought to enhance relevance and impact through contextualisation to local communities. Its importance is therefore reflected in the more recent research literature, which emphasises methods of co-design, co-production and public involvement [52. 53]. Ultimately, these methods allow for a more inclusive approach to defining self-management and ways to understand underpinning values from different standpoints.

### Strengths and limitations of the study

This study represents a novel approach to self-management research as it both acknowledges and confronts the complexity of the term itself. Rather than attempt to support or critique self-management, we regard the non-linear implications of its use as representative of the manifest turbulence in contemporary UK healthcare, and the subsequent necessity to align our exploration with this landscape. Study limitations relate to the nature of secondary analyses. Working with existing data, we were unable to elicit and collect new data specifically addressing our research questions; and we were unable to integrate a process of member checking or respondent validation in the analysis. Conversely, the use of secondary data reduced the potential for investigatory bias in the conduct of focus groups and interviews. There is scope to question the neutrality of our position, given the research interests of key members of the team. However, we have enacted a reflexive approach by openly declaring our interests; and we would assert that the distinct way, in which we have explored restrictive and productive aspects of self-management, reinforces the nonpartisan nature of our work.

## Conclusion

In conclusion, our analysis demonstrates multiple facets of power in stroke self-management, its underlying agendas, and tacit and explicit understandings which exist in real life practice. Taking a Foucauldian view, this may be understood through aspects of rhetorical, hierarchical, personal and mutual power, which are not mutually exclusive but exist in parallel. The highly routinised and structured nature of the stroke care pathway is both an explicit and implicit driver to the balance between and domination of different aspects of power, inhibiting healthcare professionals and commissioners in the degree of flexibility to interpret and enact (implement) self-management practice. We suggest that self-management practice would benefit from awareness and acknowledgment of its several nuances, theoretical and practical sensitivity towards undercurrents of power, and equity between patients, healthcare professionals and commissioners in voicing their views. This could advance the self-management discourse through greater transparency about underlying assumptions and how these might impact on practice.

## Supporting information

S1 Table Policy and practice documents included in analysis. NHS, National Health Service.
(PDF)
