# Supplementary material for: The problem with self-management: Problematising self-management and power using a Foucauldian lens in the context of stroke care and rehabilitation
Source: PLoS One. 2019 Jun 19;14(6):e0218517. doi: 10.1371/journal.pone.0218517 (PMC6584009; doi:10.1371/journal.pone.0218517)
Supplement: S1 Table — NHS, National Health Service. (PDF) [file pone.0218517.s001.pdf]

Supporting Information

The problem with self-management: Problematising self-management and power using a Foucauldian lens in the context of stroke care and rehabilitation

Simon Fletcher, Stefan Tino Kulnik, Sara Demain, Fiona Jones

S1 Table. Policy and practice documents included in analysis.

Title	Year	Domain	Reference
Long Term Health Conditions 2011 – Research Study	2011	Government	Ipsos MORI. Long Term Health Conditions 2011 – Research Study. 2011 [cited 22 March 2018] London: Crown. Available from: https://www.gov.uk/government/publications/long-term-health-conditions-2011-research-study
Long term conditions collaborative. Improving self management support.	2009	Government	Great Britain. The Scottish Government. Long term conditions collaborative. Improving self management support. Edinburgh: Crown; 2009.
National stroke strategy	2007	Government	Great Britain. Department of Health. National stroke strategy. London: Crown; 2007.
Supporting people with LTCs to self care – A guide to developing local strategies and good practice.	2006	Government	Great Britain. Department of Health. Supporting people with long term conditions to self care – A guide to developing local strategies and good practice. London: Crown; 2006.
The national service framework for long-term conditions	2005	Government	Great Britain. Department of Health. The national service framework for long-term conditions. London: Crown; 2005.
Self-care – a real choice	2005	Government	Great Britain. Department of Health. Self care – a real choice. Self care support – a practical option. London: Crown; 2005.
National service framework for coronary heart disease	2000	Government	Great Britain. Department of Health. National service framework for coronary heart disease. London: Crown; 2000.
The NHS long term plan	2019	NHS	NHS England. The NHS long term plan. 2019 [cited 20 March 2019] Available from: https://www.longtermplan.nhs.uk/publication/nhs-long-term-plan/

Next steps on the NHS Five Year Forward View	2017	NHS	NHS England. Next steps on the NHS Five Year Forward View. 2017 [cited 28 Sept 2017] Available from: https://www.england.nhs.uk/publication/next-steps-on-the-nhs-five-year-forward-view/
Commissioning guidance for rehabilitation	2016	NHS	NHS England. Commissioning guidance for rehabilitation. 2016 [cited 26 March 2018] Available from: https://www.england.nhs.uk/wp-content/uploads/2016/04/rehabilitation-comms-guid-16-17.pdf
Independent evaluation of the feasibility of using the Patient Activation Measure in the NHS in England. Summary interim report.	2016	NHS	Armstrong N, Tarrant C, Martin G, et al. Independent evaluation of the feasibility of using the Patient Activation Measure in the NHS in England. Summary interim report. 2016 [cited 14 Nov 2016] Leeds: NHS England. Available from: https://www.england.nhs.uk/wp-content/uploads/2016/04/pa-interim-report-summary.pdf
NHS England business plan	2015	NHS	NHS England. NHS England business plan. 2015 [cited 14 Nov 2016] Available from: https://www.england.nhs.uk/publications/business-plan/
Five year forward view	2014	NHS	NHS England. Five year forward view. 2014 [cited 14 Nov 2016] London: NHS England. Available from: https://www.england.nhs.uk/publication/nhs-five-year-forward-view/
National clinical guideline for stroke. Fifth edition.	2016	Clinical	Intercollegiate Stroke Working Party. National clinical guideline for stroke. 5th ed. London: Royal College of Physicians; 2016.
Stroke rehabilitation in adults. NICE guideline.	2013	Clinical	National Institute for Health and Care Excellence (NICE). Stroke rehabilitation in adults. London: NICE; 2013.
National clinical guideline for stroke. Fourth edition.	2012	Clinical	Intercollegiate Stroke Working Party. National clinical guideline for stroke. 4th ed. London: Royal College of Physicians; 2012.
National clinical guideline for stroke. Third edition.	2008	Clinical	Intercollegiate Stroke Working Party. National clinical guideline for stroke. 3rd ed. London: Royal College of Physicians; 2008.
National clinical guideline for stroke. Second edition.	2004	Clinical	Intercollegiate Stroke Working Party. National clinical guideline for stroke. 2nd ed. London: Royal College of Physicians; 2004.
A new era for stroke. Our campaign for a new national stroke strategy.	2016	Voluntary/charitable	Stroke Association. A new era for stroke. Our campaign for a new national stroke strategy. London: Stroke Association; 2016.

Spreading change. A guide to enabling the spread of person- and community-centred approaches for health and wellbeing.	2016	Voluntary/charitable	Spreading change. A guide to enabling the spread of person- and community-centred approaches for health and wellbeing. London: Health Foundation; 2016.
Supporting self-management. A guide to enabling behaviour change for health and wellbeing using person- and community-centred approaches.	2016	Voluntary/charitable	Supporting self-management. A guide to enabling behaviour change for health and wellbeing using person- and community-centred approaches. London: Health Foundation; 2016.
Together we can conquer stroke. Stroke Association strategy 2015 to 2018.	2015	Voluntary/charitable	Stroke Association. Together we can conquer stroke. Stroke Association strategy 2015 to 2018. London: Stroke Association; 2015.
Transforming our health care system. Ten priorities for commissioners.	2015	Voluntary/charitable	Naylor C, Imison C, Addicott R, et al. Transforming our health care system. Ten priorities for commissioners. 2015 [cited 14 Nov 2016] London: King's Fund. Available from: Transforming our health care system. Ten priorities for commissioners
Supporting people to manage their health. An introduction to patient activation.	2014	Voluntary/charitable	Hibbard J, Gilburt H. Supporting people to manage their health. An introduction to patient activation. London: King's Fund; 2014.
The Stroke Association manifesto 2010 to 2015.	2010	Voluntary/charitable	Stroke Association. The Stroke Association manifesto 2010 to 2015. London: Stroke Association; 2010.
NHS, National Health Service			
